# Avian ecological succession in the Amazon: A long‐term case study following experimental deforestation

**DOI:** 10.1002/ece3.5822

**Published:** 2019-11-27

**Authors:** Cameron L. Rutt, Vitek Jirinec, Mario Cohn‐Haft, William F. Laurance, Philip C Stouffer

**Affiliations:** ^1^ Biological Dynamics of Forest Fragments Project Instituto Nacional de Pesquisas da Amazônia (INPA) Manaus Brazil; ^2^ School of Renewable Natural Resources Louisiana State University and Louisiana State University AgCenter Baton Rouge LA USA; ^3^ Coleções Zoológicas – INPA Manaus Brazil; ^4^ Centre for Tropical Environmental and Sustainability Science College of Science and Engineering James Cook University Cairns Qld Australia

**Keywords:** Amazonia, colonization, deforestation, ecological species invasions, land‐use change, Neotropics, rain forest

## Abstract

1. Approximately 20% of the Brazilian Amazon has now been deforested, and the Amazon is currently experiencing the highest rates of deforestation in a decade, leading to large‐scale land‐use changes. Roads have consistently been implicated as drivers of ongoing Amazon deforestation and may act as corridors to facilitate species invasions. Long‐term data, however, are necessary to determine how ecological succession alters avian communities following deforestation and whether established roads lead to a constant influx of new species.
2. We used data across nearly 40 years from a large‐scale deforestation experiment in the central Amazon to examine the avian colonization process in a spatial and temporal framework, considering the role that roads may play in facilitating colonization.
3. Since 1979, 139 species that are not part of the original forest avifauna have been recorded, including more secondary forest species than expected based on the regional species pool. Among the 35 species considered to have colonized and become established, a disproportionate number were secondary forest birds (63%), almost all of which first appeared during the 1980s. These new residents comprise about 13% of the current community of permanent residents.
4. Widespread generalists associated with secondary forest colonized quickly following deforestation, with few new species added after the first decade, despite a stable road connection. Few species associated with riverine forest or specialized habitats colonized, despite road connection to their preferred source habitat. Colonizing species remained restricted to anthropogenic habitats and did not infiltrate old‐growth forests nor displace forest birds.
5. Deforestation and expansion of road networks into *terra firme* rainforest will continue to create degraded anthropogenic habitat. Even so, the initial pulse of colonization by nonprimary forest bird species was not the beginning of a protracted series of invasions in this study, and the process appears to be reversible by forest succession.

## Graphical abstract

We examined how nonprimary forest birds colonized a large‐scale deforestation experiment in the central Amazon across nearly 40 years, focusing on two roads that may act as corridors to facilitate species' arrivals. A disproportionate number of widespread generalists associated with secondary forest colonized quickly following deforestation, but these species remained restricted to anthropogenic habitats and did not infiltrate old‐growth forests nor displace forest birds. This suggests that the initial pulse of colonization by nonprimary forest birds was not the beginning of a protracted series of invasions in this study, and the process appears to be reversible by forest succession.

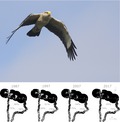

## 1 INTRODUCTION

Deforestation rates in the Amazon increased dramatically in the early 1970s, rose during the late 1990s to the highest absolute rates in the world, and accelerated once again during the early 2000s, before diminishing to the lowest rates in three decades (2012: Fearnside, 2005; INPE, 2019; Laurance, Albernaz, & Da Costa, 2001; Laurance, Cochrane, et al., 2001). In the past 4 years, however, that trend has reversed itself, with Amazon deforestation again growing to the highest rates in a decade (8,000 km^2^ in 2018; Artaxo, 2019; INPE, 2019). Roads have consistently been implicated as direct and indirect drivers of Amazon deforestation (Barber, Cochrane, Souza, & Laurance, 2014; Barni, Fearnside, & Graca, 2015; Fearnside, 2015; Fearnside & Graca, 2006; Laurance, Albernaz, et al., 2001; Laurance et al., 2002; Nepstad et al., 2001; Soares‐Filho et al., 2006). When both highways and secondary roads are taken into account, 94% of regional deforestation occurred within 5.5 km of a road; together, this network and buffer covers nearly a third (31.7%) of the Brazilian Amazon (Barber et al., 2014). Among the diverse array of deleterious effects that roads exert on the flora and fauna of tropical forests (reviewed in Laurance, Goosem, & Laurance, 2009), road networks may act as corridors to facilitate species invasions (Gascon et al., 1999; Laurance et al., 2018). However, we are not aware of any long‐term studies in Amazonia that have examined vertebrate species invasions in the context of roads and land‐use change.

For Amazonian birds, a considerable body of research has shown the toll that deforestation (including partial deforestation characterized by forest fragments) and existing roads take on the forest bird community (e.g., Ahmed et al., 2014; Develey & Stouffer, 2001; Ferraz et al., 2003; Laurance, 2004; Laurance, Stouffer, & Laurance, 2004; Lees & Peres, 2006, 2009; Mahood, Lees, & Peres, 2012; Stouffer, Johnson, Bierregaard, & Lovejoy, 2011). However, little attention has focused on these deforested landscapes and how ecological succession alters avian communities following anthropogenic change. After deforestation, early‐successional habitats could be populated by either local, preexisting forest species or colonized by foreign species from disjunct habitats, which could eventually infiltrate primary forest. Furthermore, the timing of arrival, persistence (temporary or permanent), and eventual turnover of these colonists remain poorly understood. Unfortunately, to date, most previous research has focused on short‐term, contemporary studies, which provide a static snapshot in this continual process. But due to the magnitude of Brazil's ongoing deforestation crisis, it is critically important to characterize the long‐term avifaunal changes in and adjacent to deforested regions.

To examine avian arrivals following deforestation, we chose a large‐scale experiment in the central Amazon that possesses a unique series of long‐term ornithological research—the Biological Dynamics of Forest Fragments Project (BDFFP). We employed three historical avian inventories, spread across four decades (1979–2017), to make inferences about the long‐term colonization and accumulation of species that were not part of the original forest avifauna (Cohn‐Haft, Whittaker, & Stouffer, 1997; Rutt et al., 2017; Stotz & Bierregaard, 1989). More specifically, we were interested in how patterns of avian arrivals relate to deforestation locally and along two roads leading north from the Manaus metropolitan area, a potential source for colonizing birds. Prior to the late 1970s, the region was continuous forest, but today the BDFFP represents a mosaic of regenerating second‐growth, small forest fragments, and continuous forest (Cohn‐Haft et al., 1997). Here, we (a) use the regional species pool to identify possible colonists to the BDFFP and estimate the expected proportion of arrivals by habitat type, (b) describe the chronosequence and source habitat of all birds added to the core avifauna (sensu Cohn‐Haft et al., 1997), (c) plot the location and habitat of all first detections since 1995, and (d) assess the contribution of landscape change, both locally and along two road corridors, to the process of colonization. We predict that new arrivals at the BDFFP are disproportionately represented by species from separate early‐successional habitats (e.g., second‐growth and riverine vegetation) and that these additions reflect changes in regional access via roads and not local landscape changes.

## 2 METHODS

### 2.1 Study area

**Figure 1:**
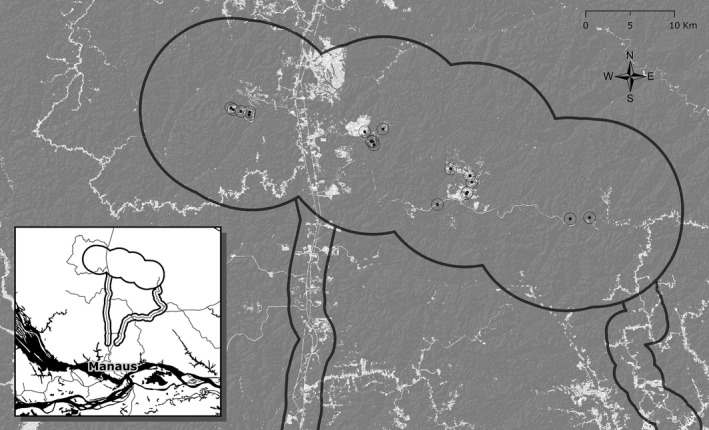
Study area, showing the Biological Dynamics of Forest Fragments Project (BDFFP; represented by a 10‐km buffer around a control reserve and three ranches), as well as 2‐km buffers around two putative avian dispersal corridors that lead north from the city of Manaus, Brazil, and the confluence of the Rio Negro and the Rio Solimões (BR‐174 on the left and AM‐010/ZF‐7 on the right). Gray background represents land cover in 2017 that was classified as closed‐canopy forest in our analyses, whereas white indicates nonforest (roads, pastures, agriculture, scrub, etc.). Symbols refer to locations where each of the most recent 19 species added to the BDFFP core avifauna was first detected. Although some have appeared in very small areas of disturbance, the vast majority of these additions are from the major disturbed areas of the ranches

The BDFFP (2°20′S, 60°W) is located ~80 km north of Manaus, Amazonas, Brazil (Figure 1). Before the project was initiated in 1979, the entire site and much of the surrounding region consisted of continuous primary *terra firme* forest. Development on three ~15,000 ha cattle ranches at the BDFFP began in the late 1970s, and forest clearing was largely complete by the mid‐1980s (Cohn‐Haft et al., 1997; Stotz & Bierregaard, 1989). These cattle ranches, however, were gradually abandoned or operated at low production levels, providing a mosaic of open pastures, second growth of various ages (from 3 to >30 years), and forest fragments embedded in a region that continues to be dominated by primary forest. To this day, regional disturbance is still minimal, except for the lands between Manaus and the BDFFP. The largest city in Amazonia, Manaus is home to >2.1 million people (July 2017), representing more than half the estimated population for the state (IBGE, 2017). Only four major roads, all paved and operational year‐round, lead outward from Manaus and connect to adjacent cities. Two of these are federal highways (BR‐174 and BR‐319) and the other two are state highways (AM‐010 and AM‐070). Here, we focus on the two paved highways that leave Manaus heading north (BR‐174 and AM‐010) toward the BDFFP (Figure 1).

### 2.2 Generating the habitat associations for the regional species pool

Neotropical birds discriminate among different vegetation types and can broadly be categorized by habitat, thus allowing us to distinguish the primary avian habitats of Amazonia (Parker, Stotz, & Fitzpatrick, 1996). *Terra firme* forests are the dominant forest type in both area and species richness (Parker et al., 1996). In Amazonia, other main vegetation types include floodplain forests (e.g., *várzea* in seasonally flooded forests along “whitewater” rivers and *igapó* along “blackwater” rivers), river island scrub, and white sand forest, each with their own distinct avifauna and local contribution to Amazonian biodiversity (Borges, 2004; Parker et al., 1996; Remsen & Parker, 1983; Rosenberg, 1990). Secondary forests, on large scales almost exclusively created by anthropogenic disturbance, are increasingly becoming an important component in Amazonia and are occupied by more broadly distributed, habitat‐generalist birds (Parker et al., 1996; Perz & Skole, 2003). Major rivers also divide closely related species, leading to areas of endemism that further increase Amazonian biodiversity (Capparella, 1991; Cracraft, 1985). Collectively, this results in a distinct regional species pool that can be characterized by habitat types and separated by interfluves, giving us the opportunity to evaluate how habitat affinities of birds in a regional species pool contribute to the avifauna of an altered site.

We first developed a list that we consider to be the “regional species pool”—those species that might reasonably be expected to occur at the study site. This seemingly arbitrary task of deciding which species are most likely was based on meeting relatively simple requirements. First, the species must have been previously recorded somewhere in the Amazon (total ~1,300 spp.), thereby assuming that the Amazonian avifauna as a whole is well‐characterized despite knowledge gaps at a regional scale. Second, species known to be limited to upland (*terra firme*) forest (see below for habitat classifications) were only included if they occur within the Guiana area of endemism, that is, north of the Amazon River and east of the lower Rio Negro. This is because these large rivers are believed to delimit distributions for *terra firme* species and, empirically, because no *terra firme* species has been found at the study site that does not also occur elsewhere within the Guiana area (see Cohn‐Haft et al., 1997 and Section 3), even if those other *terra firme* species are normally found within a few kilometers of the Guiana area, but in adjacent areas of endemism (south of the Amazon River and west of the lower Rio Negro). Third, because species from other habitats are not known to exhibit the same degree of endemism as *terra firme* birds, we relaxed our criteria, included them if previously known from within a 500 km radius if nonmigratory (resident) and a 1,000 km radius if migratory. We then curated the list by hand, adding or removing species to ensure the final product matched current knowledge. The resulting list necessarily includes all species already detected within the study area.

Using the Parker et al. (1996) databases, we added habitat associations for all birds in the regional species pool. We used the first (primary) habitat type when appropriate; however, we made adjustments, accepting secondary or tertiary habitat codes when available, if the primary code suggested the species occurred in habitat not found in central Amazonia (e.g., montane forest and temperate grassland). We collapsed these 22 categories (21 distinct habitats plus “Edge”) for the regional species pool into a more manageable seven that adequately captured habitat diversity in the immediate vicinity of the BDFFP: aquatic, primary forest, riverine, secondary forest, white sand, palm, and grassland/pasture (see Appendix A). We elected to use the term “riverine” to refer to terrestrial birds that occur in floodplain forests, river‐edge forest, and on river islands.

For those birds in the regional species pool (*n = *725), we first categorized species into two groups: those that have been recorded at the BDFFP and those that have not. Within the species that had been recorded, we categorized species as those that are part of the core forest avifauna and those that are not. The core avifauna is defined as all species that occupy primary *terra firme* forest at a relative abundance of rare, uncommon, or common (i.e., species regularly found in appropriate habitat, but not occasionally dispersing or wandering individuals; Remsen, 1994). This assemblage is a well‐characterized baseline after >35 years of ornithological coverage (Cohn‐Haft et al., 1997; Rutt et al., 2017). Those listed species that are not part of the core avifauna are presumed to have appeared following local landscape change. Three successive inventories then allowed us document the chronosequence of arriving colonists and migrants/vagrants, roughly covering the 1980s (Stotz & Bierregaard, 1989), the late 1980s to mid‐1990s (Cohn‐Haft et al., 1997), and the mid‐1990s to the present (Rutt et al., 2017). We distinguish between these two groups of noncore species by abundance, considering species that have reached a relative abundance of “uncommon” or “common” (species that occur in most or all appropriate habitats) in Cohn‐Haft et al. (1997) or Rutt et al. (2017) to have colonized and become established. Sampling has been systematic in continuous forest, fragments, and fragment borders but opportunistic in all other habitats. Taxonomy follows the South American Classification Committee (Remsen et al., 2018).

### 2.3 Location of recent additions

To verify that published habitat preferences match where a species first appears at a novel site, we plotted the approximate GPS coordinates for the first detection of each of the most recent 19 species added to the BDFFP (Figure 1; Rutt et al., 2017); no comparable raw data were available for additions before 1997. These locations were overlaid onto satellite imagery that allowed classification by coarse habitat types, which we combined with habitat descriptions from each of the species accounts in Rutt et al. (2017) to contextualize the local habitat at the time of detection.

### 2.4 Assessing long‐term changes in forest cover at the BDFFP and along two road corridors

For our purposes, we define the BDFFP study area as a 10‐km buffer around the 11 experimental fragments plus a 1,000‐ha control reserve known as Km 41 (Bierregaard, Gascon, Lovejoy, & Mesquita, 2001). For the two road corridors (BR‐174 and AM‐010/ZF‐7), we delineated 2 km buffers around each of these roads between the northern urban limits of Manaus and the southern extent of the BDFFP buffer. For all three zones, we used Landsat satellite imagery in 1987, 1997, 2007, and 2017 to quantify the extent of forest cover across 30 years. We selected cloud‐free imagery within our study area that resulted in all samples being taken during the dry season: 29 August 1987 (Landsat 5), 21 June 1997 (LS 5), 4 August 2007 (LS 5), and 30 July 2017 (LS 8). Land cover classification was conducted in GIS (ArcMap 10.5; ESRI) at 30‐m resolution for all imagery. We first generated false‐color images by combining spectral bands that create contrast between land classes of interest (bands 2, 3, 4 for LS 5, bands 3, 4, 5 for LS 8). We then classified multiband images into closed‐canopy “forest” (primary forest or mature regrowth) and “other” using ArcMap's interactive supervised classification, which employs user‐selected training samples. For “forest” training samples, we selected areas that were known to contain continuous forest that was at least 30 years old, whereas for “other” we chose bare soil, roads, clearcuts, open water, pastures, and housing. Training samples for both land cover categories were identical across the four time periods (e.g., areas that were always forest and roads). Because classified forest imagery contained many small holes, likely due to natural gap dynamics, we filled interior gaps ≤0.27 ha (3 pixels) before we calculated total forest cover.

### 2.5 Data analysis

**Figure 2:**
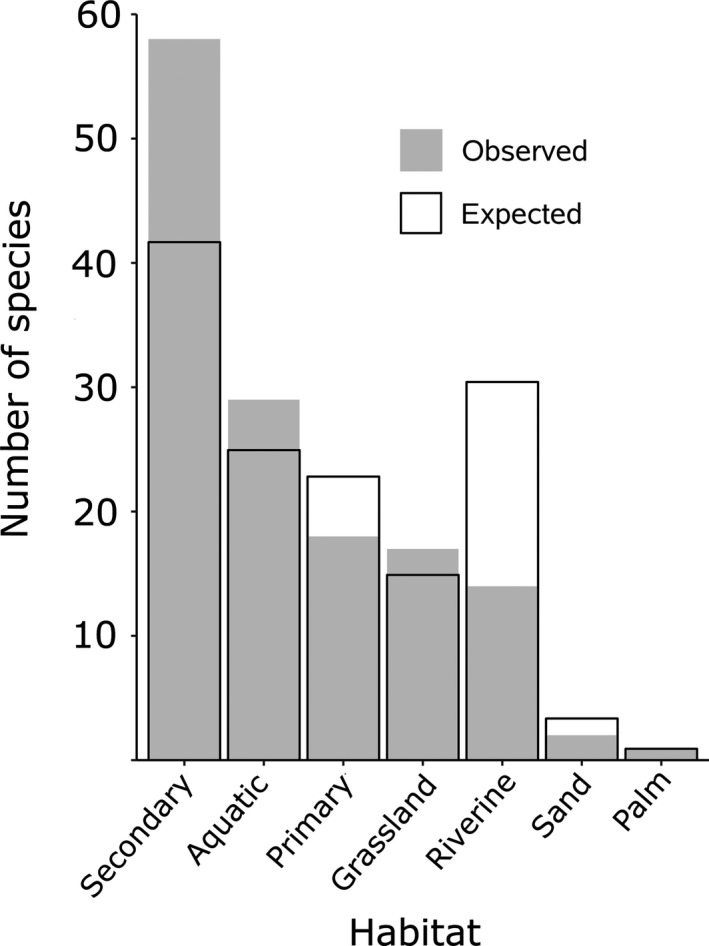
The number of observed (gray bars) and expected (empty bars) bird species per habitat added to the core avifauna at the Biological Dynamics of Forest Fragments Project (BDFFP) in the state of Amazonas, Brazil. Expected proportions were derived by assuming that species would filter passively in numbers proportional to the habitat associations of the Manaus regional species pool and, together, would sum to 139 species, the total number of birds added to the core avifauna of the BDFFP

To determine whether habitat associations of colonists and migrants/vagrants are disproportional to habitat associations of available birds in the regional species pool (i.e., excluding the core avifauna), we used *G* tests of independence. We similarly performed *G* tests to determine whether habitat associations of noncore species that appeared early (1979–86) and late (1987–2017) differed significantly from the regional species pool. If an overall *G* test was significant, we then ran post hoc tests with a Bonferroni correction—each nominal variable against the sum of all others (additional 2 × 2 contingency tables)—to identify habitat(s) that were disproportionately contributing colonists or additions (Figure 2).

## 3 RESULTS

### 3.1 Community structure and habitat associations

Our regional species pool of 725 species included more than half of all known Amazonian bird species (see Rutt, Jirinec, Cohn‐Haft, Laurance, & Stouffer, 2019). From that pool, 407 (56%) have been recorded at the BDFFP (Rutt et al., 2017). The core avifauna at the BDFFP typifies the forest community prior to disturbance and comprises 268 species (Rutt et al., 2017). Since 1979, 139 species that are not a part of the core avifauna have been recorded at the BDFFP: 85 were added by 1986, another 35 by 1994, and the final 19 by 2017 (Table 1). The vast majority of these additions are considered rare or vagrants at the BDFFP (99 species; 71% of additions) or are regular austral or boreal migrants (5 species; 4%). We considered the remaining 35 species to be established permanent residents (Table 2). We found no species endemic to areas west of the Rio Negro or south of the Amazon River. All primary *terra firme* forest birds at the BDFFP are widespread in the Guianan region.

**Table 1:** Time period of first detection by habitat association for bird species added to the core avifauna at the Biological Dynamics of Forest Fragments Project (BDFFP) in the state of Amazonas, Brazil

Habitat	Total species possible	Number of new species recorded (colonized)			Species never recorded
		1979–1986	1987–1994	1995–2017	
Aquatic	82	21 (2)	6	2	53
Secondary	137	**38 (20)**	13 **(2)**	7	79
Primary	75	10 (2)	4	4	57
Sand	11	1 (1)	0	1	9
Palm	3	1	0	0	2
Riverine	100	**3 (2)**	7	4	86
Grassland	49	11 (6)	5	1	32
Total	457^a^	85 (33)	35 (2)	19	318
% of new species		61%	25%	14%	
% of colonists		94%	6%	0%	

Note

Total species possible enumerates the regional species pool (minus the already identified core avifauna; see Section 2) and the final column those that have never been recorded at the BDFFP. Numbers in parentheses designate how many species of a particular habitat colonized during that interval; the balance refers to migrants and vagrants.

Bold cell values indicate statistically significant deviations from expected values given the total species possible (first column).

a 457 = 725 (regional species pool) – 268 (core avifauna).

**Table 2:** Thirty‐five bird species that colonized the Biological Dynamics of Forest Fragments Project in the state of Amazonas, Brazil, along with the interval during which each species was first detected on site and its habitat affiliation according to the Parker et al. (1996) databases

Scientific name	English name	1986	1994	2017	Habitat
*Ortalis motmot*	Variable Chachalaca	x	x	x	Secondary
*Tachybaptus dominicus*	Least Grebe	x	x	x	Aquatic
*Leptotila verreauxi*	White‐tipped Dove	x	x	x	Secondary
*Crotophaga ani*	Smooth‐billed Ani	x	x	x	Secondary
*Piaya cayana*	Squirrel Cuckoo	x	x	x	Primary
*Nyctidromus albicollis*	Common Pauraque	x	x	x	Secondary
*Anurolimnas viridis*	Russet‐crowned Crake	x	x	x	Grassland
*Jacana jacana*	Wattled Jacana	x	x	x	Aquatic
*Cathartes aura*	Turkey Vulture	x	x	x	Grassland
*Coragyps atratus*	Black Vulture	x	x	x	Secondary
*Buteogallus meridionalis*	Savanna Hawk	x	x	x	Grassland
*Rupornis magnirostris*	Roadside Hawk	x	x	x	Secondary
*Buteo nitidus*	Gray‐lined Hawk	x	x	x	Secondary
*Buteo brachyurus*	Short‐tailed Hawk	x	x	x	Primary
*Milvago chimachima*	Yellow‐headed Caracara	x	x	x	Grassland
*Thamnophilus punctatus*	Northern Slaty‐Antshrike		x	x	Secondary
*Cercomacroides tyrannina*	Dusky Antbird	x	x	x	Secondary
*Myiozetetes cayanensis*	Rusty‐margined Flycatcher	x	x	x	Secondary
*Empidonomus varius*	Variegated Flycatcher	x	x	x	Secondary
*Tyrannus melancholicus*	Tropical Kingbird	x	x	x	Secondary
*Tyrannus savana*	Fork‐tailed Flycatcher	x	x	x	Grassland
*Myiarchus ferox*	Short‐crested Flycatcher	x	x	x	Riverine
*Neopelma chrysocephalum*	Saffron‐crested Tyrant‐Manakin	x	x	x	Sand
*Manacus manacus*	White‐bearded Manakin		x	x	Secondary
*Stelgidopteryx ruficollis*	Southern Rough‐winged Swallow	x	x	x	Secondary
*Troglodytes aedon*	House Wren	x	x	x	Secondary
*Volatinia jacarina*	Blue‐black Grassquit	x	x	x	Secondary
*Ramphocelus carbo*	Silver‐beaked Tanager	x	x	x	Secondary
*Sporophila castaneiventris*	Chestnut‐bellied Seedeater	x	x	x	Secondary
*Sporophila angolensis*	Chestnut‐bellied Seed‐Finch	x	x	x	Secondary
*Thraupis episcopus*	Blue‐gray Tanager	x	x	x	Secondary
*Thraupis palmarum*	Palm Tanager	x	x	x	Secondary
*Ammodramus aurifrons*	Yellow‐browed Sparrow	x	x	x	Riverine
*Molothrus bonariensis*	Shiny Cowbird	x	x	x	Secondary
*Sturnella militaris*	Red‐breasted Meadowlark	x	x	x	Grassland

Note

A species was considered to have colonized and become established if it was not a part of the original core avifauna and it reached a relative abundance of “uncommon” or “common” in 1994 or 2017 (Cohn‐Haft et al., 1997; Rutt et al., 2017).

Taken altogether, habitat associations of the 139 species of colonists and migrants/vagrants at the BDFFP were not representative of habitat associations for available birds in the regional species pool (*G* = 27.11, *df* = 6, *p* < .001, Figure 2). Excluding those habitats with very few species (sand and palm), post hoc tests with a Bonferroni correction (*p* = .01) revealed that more secondary forest species (*G* = 12.28, *df* = 1, *p* < .001) and fewer riverine species (*G* = 18.87, *df* = 1, *p* < .001; Figure 2) appeared than would have been expected from the regional species pool. The pattern was identical in the restricted subset of colonists (*G* = 26.38, *df* = 6, *p < *.001), with more secondary forest species (*G* = 17.91, *df* = 1, *p* < .001) and fewer riverine species (*G* = 7.56, *df* = 1, *p* = .006) than predicted by the regional species pool. This difference in habitat association, however, was only evident for the 85 species added during the 1980s (Table 1; *G* = 35.40, *df* = 6, *p* < .001) and was not significant for the subsequent 54 additions that accumulated from the late 1980s through the 2000s (*G* = 4.41, *df* = 6, *p* = .62). Only during the 1980s did more secondary forest species (*G* = 10.04, *df* = 1, *p* = .002) and fewer riverine species (*G* = 27.83, *df* = 1, *p* < .001) appear than were expected from the regional species pool.

### 3.2 Location of recent additions

With only one exception, all of the 19 species whose preferred habitat can be found at the BDFFP (i.e., primary forest, secondary forest, or aquatic) were first detected in that habitat. The lone exception was Scaled Pigeon (*Patagioenas speciosa*; primary forest), which was first detected in mature secondary forest; however, this species' local and published habitat affinities actually include a variety of shorter and sparser forests, and it does not typically occupy primary forest here (see species account in Rutt et al., 2017). Species that Parker et al. (1996) classified as grassland (Upland Sandpiper [*Bartramia longicauda*]), riverine (Cinnamon Attila [*Attila cinnamomeus*], White‐throated Kingbird [*Tyrannus albogularis*]), and sand (Yellow‐crested Manakin [*Heterocercus flavivertex*]) birds—habitats not present at the BDFFP—first appeared in the closest on‐site analogs: pasture, a moriche palm (*Mauritia flexuosa*) swamp and forest pond, and stunted secondary forest, respectively. Two additional riverine species (Black‐chinned Antbird [*Hypocnemoides melanopogon*] and Yellow‐rumped Cacique [*Cacicus cela*]) were found at primary forest sites, but one was in a small camp clearing and the other in a small (10‐ha) forest fragment, suggesting association with local disturbance.

### 3.3 Temporal landscape changes

**Figure 3:**
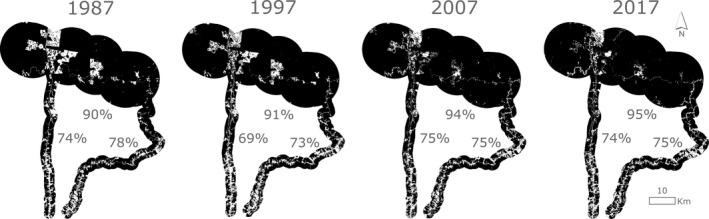
Results of land cover classification as closed‐canopy forest (primary forest or mature regrowth; black) and nonforest (white) using Landsat imagery across 30 years at the Biological Dynamics of Forest Fragments Project (BDFFP) and along two highways that connect the city of Manaus, Brazil, to the BDFFP. The percent of forest within the BDFFP and along each of the two corridors (BR‐174 to the west and AM‐010/ZF‐7 to the east) is illustrated during all four time periods

As delineated by our binary landscape classification, the BDFFP has been predominantly covered by closed‐canopy forest across all four time periods (90.0%–94.8%; Figure 3), becoming more forested from 1987 to 2017. Although the majority of the two road buffers was also comprised of closed‐canopy forest (an average of 73.1% along BR‐174 and 75.5% along AM‐010/ZF‐7), nonforest habitat was much more uniformly distributed and prevalent, remaining between 21.6% and 30.6% of the total area of each road buffer during all four time periods. There were no clear temporal trends in the extent of forest cover along the two road corridors, as both had similar proportions of closed‐canopy forest in 1987 and 2017 (Figure 3).

## 4 DISCUSSION

Our long‐term data allow us to describe the accumulation of novel species into an Amazon forest bird community following deforestation. In all, 139 species that are not part of the core avifauna have been added during the past ~40 years (1979–2017), representing 34% of the present BDFFP list (Rutt et al., 2017). Thirty‐five species are considered to have colonized and since become established at the BDFFP, a nontrivial addition to the local species assemblage—13% of the core avifauna (Rutt et al., 2017). Furthermore, because we can relate the detection of these species across time as well as to large‐scale temporal landscape changes, this study offers insight into the process of avian colonization and ecological species invasions (hereafter, “invasions”).

### 4.1 Invasions happen quickly

Despite relatively unchanging land cover at both the BDFFP and along two road corridors, novel species arrived quickly following deforestation and creation of pastures. Most additions to the original forest avifauna (61%; 85/139) were detected during the 1980s. Similarly, almost all colonists (94%; 33/35)—species that presumably established new breeding populations—first appeared during the 1980s.

### 4.2 Colonists were mostly widespread generalist species

Ubiquitous, widespread generalists associated with secondary forest appeared in greater numbers than were expected by passive filtering according to the regional species pool. Furthermore, of the noncore species that colonized and became established at the BDFFP, a disproportionate number (22 species; 63%) are classified as secondary forest birds (Table 2). Assuming that all species evolved in natural, nonanthropic habitats, the bulk of these habitat generalists now able to exploit anthropogenic second growth likely originated from river‐edge habitat in the region (Terborgh & Weske, 1969). As classified here, however, riverine species are largely comprised of more habitat specialists, and fewer riverine species appeared at the BDFFP or colonized than were expected by chance. Thus, it seems that the most specialized riverine birds (true floodplain forest species and river island obligates) rarely disperse far inland, even along river‐like road disturbances, or colonize new sites such as the BDFFP. Further evidence of this is the fact that the avifauna of the city of Manaus is dominated by floodplain forest birds (M. Cohn‐Haft, pers. obs.), but many of these have not progressed farther inland nor reached the BDFFP, even though the city would seem to be a reasonable source for colonizing birds. Instead, primarily generalist species that are today associated with secondary forest actively dispersed into and colonized the BDFFP. Furthermore, despite >35 years of ornithological coverage, we never detected a single forest species from adjacent areas of endemism (west of the Rio Negro or south of the Amazon River).

### 4.3 Exotic species did not colonize

Interestingly, no truly exotic species (non‐Amazonian or non‐South American) have become established in our study area. The only such species found anywhere nearby are Cattle Egrets (*Bubulcus ibis*), Rock Pigeons (*Columba livia*), House Sparrows (*Passer domesticus*), and Common Waxbills (*Estrilda astrild*). The egrets are known as accidental at our sites and have probably not become established simply because the cattle ranches have all failed (Laurance et al., 2018). The other three species are present in the city of Manaus (pers. obs., Borges, Pacheco, & Whittaker, 1996), but have not been found away from dense human populations. This appears to attest to the resistance of Amazonian primary forest to invasion by exotic species, as well as the apparent resistance of disturbed, secondary forests.

### 4.4 Colonizers are not infiltrating old‐growth forests

Those species that have colonized the BDFFP only rarely penetrate primary forest or the interior of large fragments, and none have colonized these habitats. Out of the 35 colonists, 16 (46%) have been captured at least once during long‐term bird banding effort, for a total of 656 captures in our >69,000 capture dataset. However, most of these records are from very small forest fragments (1‐ha) or from nets placed along the border of larger fragments. Excluding captures within ~100 m of a forest border at all other sites leaves only 44 captures of 7 species (predominantly White‐bearded Manakin [*Manacus manacus*], House Wren [*Troglodytes aedon*], Silver‐beaked Tanager [*Ramphocelus carbo*], and Chestnut‐bellied Seed‐Finch [*Sporophila angolensis*]). Furthermore, only four of 35 species (Variable Chachalaca [*Ortalis motmot*], Common Pauraque [*Nyctidromus albicollis*], Silver‐beaked Tanager, and Blue‐gray Tanager [*Thraupis episcopus*]) were detected eight times in a 100‐ha continuous forest plot during an intensive whole‐community inventory (Johnson, Stouffer, & Vargas, 2011). Thus, invading birds largely represent nonforest taxa restricted to anthropogenic habitats in the matrix and rarely penetrate closed‐canopy forests, consistent with the earlier suggestion that intact rainforests are generally resistant to species invasions (Laurance & Bierregaard, 1997). Therefore, we believe that these additions and invaders have a minimal ecological impact on the intact forest (e.g., seed dispersal of pioneer plant species and nest parasitism), although they could be playing nontrivial roles in matrix and disturbed habitats, including the potential introduction of novel pathogens (Altizer, Bartel, & Han, 2011). Similarly, no primary forest birds colonized early‐successional habitat following disturbance.

### 4.5 Some species are still trickling in whereas others are retreating in response to forest succession

The appearance of novel species at the BDFFP is far from random and includes many species that were predicted to eventually arrive (Cohn‐Haft et al., 1997). Despite considerably less fieldwork at Reserva Ducke—near the juncture of BR‐174 and AM‐010 along the outskirts of Manaus—Willis (1977) found 30 species not reported at the BDFFP during the first inventory (Stotz & Bierregaard, 1989). Within the following decade, however, 14 of those 30 species had appeared at the BDFFP (Cohn‐Haft et al., 1997), and another six were detected between 1995 and 2017 (Rutt et al., 2017). Additional secondary forest species are still trickling in and may be in the early stages of colonization (Tropical Screech‐Owl [*Megascops choliba*], Yellow‐bellied Elaenia [*Elaenia flavogaster*], Boat‐billed Flycatcher [*Megarynchus pitangua*], Brown‐crested Flycatcher [*Myiarchus tyrannulus*], and White‐lined Tanager [*Tachyphonus rufus*]). At the same time, a number of established colonists have become rarer as their preferred habitat at the BDFFP decreased between 1997 and 2017 (e.g., ground‐doves [*Columbina* spp.], House Wren, Yellow‐browed Sparrow [*Ammodramus aurifrons*], Blue‐black Grassquit [*Volatinia jacarina*], Chestnut‐bellied Seedeater [*Sporophila castaneiventris*], *Thraupis* spp., and Red‐breasted Meadowlark [*Sturnella militaris*]). Capture data reveal similar trends; for instance, there were 36 captures of House Wren between 1981 and 1993, but none thereafter. Similarly, Silver‐beaked Tanager was captured 232 times during that interval and only 21 times thereafter. Many of these early‐successional species were previously characterized as common and are obvious and familiar avian components around human habitation in the region. Although early‐successional species appear to be largely declining, however, some secondary forest species seem to be increasing (e.g., Dusky Antbird [*Cercomacroides tyrannina*], White‐bearded Manakin, and Buff‐throated Saltator [*Saltator maximus*]).

### 4.6 Are roads to blame?

Given that roads are both direct and indirect drivers of Amazonian deforestation (Barber et al., 2014; Barni et al., 2015; Fearnside, 2015; Fearnside & Graca, 2006; Laurance et al., 2002; Laurance, Cochrane, et al., 2001; Nepstad et al., 2001; Soares‐Filho et al., 2006), it is apparent that roads are promoting species invasions both directly (as invasion corridors) and indirectly (by promoting land‐use changes). Cohn‐Haft et al. (1997) first proposed the idea that roads visually resemble rivers, including adjacent successional vegetation, and may serve as biological conveyor belts to transport species from extensive areas of disturbance near Manaus into previously undisturbed rainforest. Roads have been specifically implicated as catalysts for some invasions and range expansions in Amazonia, in particular, the advancement of House Sparrows, a species exclusively associated with humans (Smith, 1973, 1980). At our site, Cohn‐Haft et al. (1997) described watching Swallow‐winged Puffbirds (*Chelidoptera tenebrosa*) progress incrementally farther north from Manaus along BR‐174 until it was eventually detected (1991) at the BDFFP itself. We cannot confirm that roads have been the conduit for colonization, although the continuous extension of disturbed vegetation they have consistently presented over time is likely to have benefited many of the colonizing species we detected. On the other hand, in spite of a river‐like disturbance corridor leading outward from the city of Manaus, long‐range dispersal of true floodplain forest specialists has been very limited.

### 4.7 Natural habitat succession can remove potential colonists

Our data also suggest that if land abandonment and forest recovery are shielded from further disturbance and allowed to proceed unimpeded—especially while sufficiently connected to primary forest—regenerating secondary forests offer another advantage: the ability to weed out invading species over time. Our data indicate that where forest cover has recuperated over time, the presence of early‐successional bird species has diminished. This is similar to the well‐documented trend of increasing rarity of open‐country birds with the reforestation and afforestation of the eastern United States (Askins, 2000; Brennan & Kuvlesky, 2005). Although debate continues about the conservation value of secondary forests (Brook, Bradshaw, Koh, & Sodhi, 2006; Wright & Muller‐Landau, 2006a, 2006b), the extent of secondary forests in the Brazilian Amazon is increasing (Neeff, Lucas, dos Santos, Brondizio, & Freitas, 2006; Perz & Skole, 2003). We believe that natural forest regeneration can further serve as an effective tool to eliminate new, distinct communities of invading colonists, providing further opportunity for the original forest avifauna to recover.

### 4.8 Species richness alone is an inappropriate indicator of habitat quality for partially disturbed sites

Although a commonly used metric in conservation assessments, our synthesis of these historical avian inventories also illustrates how species richness itself fails to capture landscape degradation. Total species richness increased by >100 species over the past nearly four decades—due to the foreign contribution of predominantly secondary forest birds—despite the project area losing ~10% of primary forest when the cattle ranches were clearcut beginning in 1979. Of course, this would be expected with the appearance of novel habitats and their associated avifauna, but we nonetheless believe this is worth highlighting because a greater number of species is typically synonymous with greater conservation value. The apparent increase in species richness, however, is inconsequential, as regional conservation measures should be aimed at species dependent on primary forest (habitat specialists), not widespread habitat generalists able to exploit anthropogenic disturbances. These latter species are simply a natural byproduct of disturbance and ecological succession in degraded landscapes. Thus, it is critical that we guard against these sorts of singular species richness assessments and instead focus on the constituent members of an identified community.

## 5 CONCLUSIONS

Approximately 20% of the Brazilian Amazon has now been deforested (Artaxo, 2019), including what amounts to a region of deforestation larger than the state of California since 1988 (INPE, 2019). Furthermore, the recent return to increasing deforestation rates seems likely to continue as a newly appointed administration led by President Jair Bolsonaro (inaugurated 1 January 2019) threatens to expand mining, pasture, and agricultural activities in the Amazon (Artaxo, 2019; Escobar, 2018). Amazon road networks are also anticipated to continue expanding (Ahmed, Ewers, & Smith, 2013; Ahmed et al., 2014; Ahmed, Souza, Riberio, & Ewers, 2013; Laurance et al., 2014). A recent analysis across the entire Brazilian Amazon found that, on average, nearly 17,000 km of roads were added every year between 2004 and 2007 (Ahmed, Souza, et al., 2013). In all, more than 260,000 km of roads (>70% unofficial or illegal) crisscross the Brazilian Amazon, enough to stretch more than two‐thirds the distance between the Earth and the moon (Barber et al., 2014). Given continued deforestation and habitat degradation in the Amazon, it is imperative that conservationists not only describe the quality of anthropogenic habitat for forest‐dependent birds, but also the biotic interchange and potential species interactions cultivated by these distinct habitats. Our unique, site‐specific data from one of the very few long‐term study areas in the Amazon provide an important benchmark to describe the processes of avian species invasions and ecological succession, as well as the separation of these anthropogenic avian communities.

We believe that the pattern of species accumulation and colonization of widespread generalists that we describe here is likely generalizable across Amazonia following deforestation, agricultural use, and eventual abandonment. The addition and establishment of 35 bird species to a once undisturbed tract of rainforest over about 40 years offers some of the strongest, large‐scale documentation of vertebrate species invasions in Amazonia following anthropogenic disturbance. The dire consequences of deforestation for primary forest birds, however, cannot be overlooked. Following deforestation, the two coexisting local communities—primary forest and pasture—largely remained segregated, and those new colonists did not invade intact habitat nor displace forest birds. Only a long‐term study site such as the BDFFP would be capable of describing this protracted process and monitoring changing communities over time, both of which would remain hidden in short‐term research. It will, however, take a much longer period of time to detect the possibility of eventual recovery and stability of the original avian community in these degraded habitats (Powell, 2013). Finally, we look forward to the results of future long‐term research to determine whether our results are applicable across other taxa and regions in Amazonia.

## CONFLICT OF INTEREST

None declared.

## AUTHOR CONTRIBUTIONS

CLR and PCS conceived of the study. MCH and CLR generated the regional species pool. VJ conducted the temporal landscape analysis. CLR and PCS analyzed the contingency tables. All authors contributed to the interpretation of the results. CLR wrote the manuscript with support and input from all coauthors.

## Data Availability

A list of all species that we included in the regional species pool is archived at the Dryad Digital Repository https://doi.org/10.5061/dryad.xpnvx0kb2. We annotated this list with the habitat associations for all species and designated which of these have already been detected at the BDFFP, as well as the more restricted subset that we consider to be members of the core avifauna.

## APPENDIX A

The seven categories that characterize habitat diversity at the Biological Dynamics of Forest Fragments Project (BDFFP) and the 22 categories that these were derived from in the Parker et al. databases (1996).

**Table:**

BDFFP category	Parker et al. category
Aquatic	Freshwater marshes (A1)
	Saltwater/brackish marshes (A2)
	Coastal sand beaches/mudflats (A3)
	Riverine sand beaches (A5)
	Freshwater lakes (A6)
	Rivers (A8)
	Streams (A9)
	Coastal waters (A11)
Primary	Tropical lowland evergreen forest (F1)
Riverine	Flooded tropical evergreen forest (F2)
	River‐edge forest (F3)
	River island scrub (N12)
Sand	White sand forest (F12)
Palm	Palm forest (F13)
Grassland/Pasture	Campo grasslands (N5)
	Low, seasonally wet grassland (N6)
	Pastures/agricultural lands (N13)
Secondary	Tropical deciduous forest (F7)
	Secondary forest (F15)
	Arid lowland scrub (N1)
	Second‐growth scrub (N14)
	Edge (E)
